# Computing the Entropy Measures for the Line Graphs of Some Chemical Networks

**DOI:** 10.1155/2022/2006574

**Published:** 2022-10-06

**Authors:** Muhammad Farhan Hanif, Hasan Mahmood, Shazia Manzoor, Fikre Bogale Petros

**Affiliations:** ^1^Abdus Salam School of Mathematical Sciences, Government College University, Lahore, Pakistan; ^2^Department of Mathematics, Government College University, Lahore, Pakistan; ^3^Department of Mathematics, COMSATS University Islamabad, Lahore Campus, Pakistan; ^4^Department of Mathematics, Addis Ababa University, Addis Ababa, Ethiopia

## Abstract

Chemical Graph entropy plays a significant role to measure the complexity of chemical structures. It has explicit chemical uses in chemistry, biology, and information sciences. A molecular structure of a compound consists of many atoms. Especially, the hydrocarbons is a chemical compound that consists of carbon and hydrogen atoms. In this article, we discussed the concept of subdivision of chemical graphs and their corresponding line chemical graphs. More preciously, we discuss the properties of chemical graph entropies and then constructed the chemical structures namely triangular benzenoid, hexagonal parallelogram, and zigzag edge coronoid fused with starphene. Also, we estimated the degree-based entropies with the help of line graphs of the subdivision of above mentioned chemical graphs.

## 1. Introduction

Mathematical chemistry is a field of theoretical chemistry that uses mathematical approaches to discuss molecular structure without necessarily referring to quantum mechanics [1]. Chemical Graph Theory is a branch of mathematical chemistry where a chemical phenomenon is theoretically described using graph theory [2, 3]. The growth of organic disciplines has been aided by Chemical Graph Theory [4, 5]. In mathematical chemistry, graph invariants or topological indices are numeric quantities that describe various essential features of organic components and are produced from an analogous molecular graph [6, 7]. Degree-based indices are among the topological indices used to predict bioactivity, boiling point, draining energy, stability, and physico-chemical properties of certain chemical compounds [8, 9]. Due to their chemical applications, these indices have significant role in theoretical chemistry. Zhang et al. [10–12] discuss the topological indices of generalized bridge molecular graphs, Carbon Nanotubes and product of chemical graphs. Zhang et al. [13–15] provided the physical analysis of heat for formation and entropy of Ceria Oxide. For further study about indices, see [16, 17].Shannon [18] originated the conception of information entropy in communication theory. However, it was later discovered as a quantity that applied to all things with a set nature [19, 20], including molecular graphs [21–23]. In chemistry, information entropy is now used in two modes. Firstly, it is a structural descriptor for assessing the complexity of chemical structures [24]. Information entropy is useful in this regard for connecting structural and physico-chemical features [25], numerically distinguishing isomers of organic molecules [26], and classifying natural products and synthetic chemicals [27, 28]. The physico-chemical sounding of information entropy is a different mode of application. As a result, Terenteva and Kobozev demonstrated its utility in analyzing physico-chemical processes that simulate information transmission [29]. Zhdanov [30] used entropy values to study organic compound chemical processes. The information entropy is defined as:

(1) $$\begin{array}{c} ENT_{\psi }\left(F\right)=-\overset{q}{\underset{i=1}{\sum }}N_{i}\frac{\Lambda \left(l_{i}m_{i}\right)}{In}\log\frac{\Lambda \left(l_{i}m_{i}\right)}{In}\text{,}\\ =\log\;\left(In\right)-\frac{1}{In}\overset{q}{\underset{i=1}{\sum }}N_{i}\Lambda \left(l_{i}m_{i}\right)\log\;\Lambda \left(l_{i}m_{i}\right)\text{.} \end{array}$$

Here, the logarithm is considered to be with base *e* while *ℱ*_*V*_ , *ℱ*_*E*_ and Λ(*lm*) represent the vertex set, the edge set and the edge weight of the edge (*lm*) in Λ. Many graph entropies have been calculated in the literature utilising characteristic polynomials, vertices degree, and graph order [31–34]. Graph entropies, which are based on independent sets, matchings, and the degree of vertices [35], have been estimated in recent years. Dehmer and Mowshowits proposed several graph complexity and Hosoya entropy relationships [23, 32, 36, 37]. For further study, see [19, 21, 38–42, 59, 60].The graph *ℱ* is structured into ordered pairs, with one object being referred to as a vertex set (*ℱ*_*V*_) and the other as an edge set (*ℱ*_*E*_), and these vertices and edges being connected. When two vertices of *ℱ* share an edge, they are said to be neighboring. The sum of the degrees of all neighboring vertices of *l* is denoted by *A*_*l*_, and the degree of a vertex *l* is represented by $\overset{\wedge }{ℵ}\left(l\right)$. By replacing each of *S*(*ℱ*)'s edges with a path of length two, the subdivision graph *S*(*ℱ*) is formed. The line graph is denoted by the symbol *L*(*ℱ*) in which |*V*(*L*(*ℱ*))| = |*E*(*ℱ*)| and two vertices of *L*(*ℱ*) are adjacent iff their corresponding edges share a common end points in *ℱ*.

### 1.1. Randić Entropy [43, 44]

If $\Lambda \left(lm\right)={\left(\overset{\wedge }{ℵ}\left(l\right)\times \overset{\wedge }{ℵ}\left(m\right)\right)}^{\alpha },with\;\alpha =1,-1,1/2,-1/2$, then

(2) $$\begin{array}{c} \underset{lm\in F_{E}}{\sum }\Lambda \left(lm\right)=\underset{lm\in F_{E}}{\sum }{\left(\overset{\wedge }{ℵ}\left(l\right)\times \overset{\wedge }{ℵ}\left(m\right)\right)}^{\alpha }=R_{\alpha }. \end{array}$$

Now (1) represent the Randi$\overset{\prime }{c}$ Entropy.

(3) $$\begin{array}{c} ENT_{R_{\alpha }}\left(F\right)=\log\left(R_{\alpha }\right)-\frac{1}{\left(R_{\alpha }\right)}\overset{q}{\underset{i=1}{\sum }}\underset{lm\in F_{E_{i}}}{\sum }\left[{\left(\overset{\wedge }{ℵ}\left(l\right)\times \overset{\wedge }{ℵ}\left(m\right)\right)}^{\alpha }\right]\log\left[{\left(\overset{\wedge }{ℵ}\left(l\right)\times \overset{\wedge }{ℵ}\left(m\right)\right)}^{\alpha }\right]\text{.} \end{array}$$

### 1.2. Atom Bond Connectivity Entropy [45]

If $\Lambda \left(lm\right)=\sqrt{\overset{\wedge }{ℵ}\left(l\right)\times \overset{\wedge }{ℵ}\left(m\right)-2/\overset{\wedge }{ℵ}\left(l\right)\times \overset{\wedge }{ℵ}\left(m\right)}$, then

(4) $$\begin{array}{c} \underset{lm\in F_{E}}{\sum }\Lambda \left(lm\right)=\underset{lm\in F_{E}}{\sum }\sqrt{\frac{\overset{\wedge }{ℵ}\left(l\right)+\overset{\wedge }{ℵ}\left(m\right)-2}{\overset{\wedge }{ℵ}\left(l\right)\times \overset{\wedge }{ℵ}\left(m\right)}}=ABC\left(F\right). \end{array}$$

Thus (1) is converted in the following form:

(5) $$\begin{array}{c} ENT_{ABC}\left(F\right)=\log\;\left(ABC\right)-\frac{1}{\left(ABC\right)}\overset{q}{\underset{i=1}{\sum }}\underset{lm\in F_{E_{i}}}{\sum }\left[\sqrt{\frac{\overset{\wedge }{ℵ}\left(l\right)+\overset{\wedge }{ℵ}\left(m\right)-2}{\overset{\wedge }{ℵ}\left(l\right)\times \overset{\wedge }{ℵ}\left(m\right)}}\right]\log\left[\sqrt{\frac{\overset{\wedge }{ℵ}\left(l\right)+\overset{\wedge }{ℵ}\left(m\right)-2}{\overset{\wedge }{ℵ}\left(l\right)\times \overset{\wedge }{ℵ}\left(m\right)}}\right]\text{.} \end{array}$$

### 1.3. The Geometric Arithmetic Entropy [43, 44]

If $\Lambda \left(lm\right)=2\sqrt{\overset{\wedge }{ℵ}\left(l\right)\times \overset{\wedge }{ℵ}\left(m\right)}/\overset{\wedge }{ℵ}\left(l\right)+\overset{\wedge }{ℵ}\left(m\right)$, then

(6) $$\begin{array}{c} \underset{lm\in F_{E}}{\sum }\Lambda \left(lm\right)=\underset{lm\in F_{E}}{\sum }\frac{2\sqrt{\overset{\wedge }{ℵ}\left(l\right)\times \overset{\wedge }{ℵ}\left(m\right)}}{\overset{\wedge }{ℵ}\left(l\right)+\overset{\wedge }{ℵ}\left(m\right)}=GA\left(F\right)\text{.} \end{array}$$

Now (1) takes the form as given below.

(7) $$\begin{array}{c} ENT_{GA}\left(F\right)=\log\;\left(GA\right)-\frac{1}{\left(GA\right)}\overset{q}{\underset{i=1}{\sum }}\underset{lm\in F_{E_{i}}}{\sum }\left[\frac{2\sqrt{\overset{\wedge }{ℵ}\left(l\right)\times \overset{\wedge }{ℵ}\left(m\right)}}{\overset{\wedge }{ℵ}\left(l\right)+\overset{\wedge }{ℵ}\left(m\right)}\right]\log\left[\frac{2\sqrt{\overset{\wedge }{ℵ}\left(l\right)\times \overset{\wedge }{ℵ}\left(m\right)}}{\overset{\wedge }{ℵ}\left(l\right)+\overset{\wedge }{ℵ}\left(m\right)}\right]\text{.} \end{array}$$

### 1.4. The Fourth Atom Bond Connectivity Entropy [35]

If $\Lambda \left(lm\right)=\sqrt{A_{l}+A_{m}-2/A_{l}A_{m}}$, then

(8) $$\begin{array}{c} \underset{lm\in E\left(F\right)}{\sum }\Lambda \left(lm\right)=\underset{lm\in E\left(F\right)}{\sum }\sqrt{\frac{A_{l}+A_{m}-2}{A_{l}A_{m}}}=ABC_{4}\left(F\right)\text{.} \end{array}$$

Now (1) converted in the following form as:

(9) $$\begin{array}{c} ENT_{ABC_{4}}\left(F\right)=\left.\log\;\left(ABC_{4}\left(F\right)\right)-\frac{1}{\left(ABC_{4}\left(F\right)\right)}\overset{q}{\underset{i=1}{\sum }}\underset{lm\in E_{i}\left(F\right)}{\sum }\log{\left[\sqrt{\frac{A_{l}+A_{m}-2}{A_{l}A_{m}}}\right]}^{\left[\sqrt{A_{l}+A_{m}-2/A_{l}A_{m}}\right]}\right]\text{.} \end{array}$$

### 1.5. The Fifth Geometric Arithmetic Entropy [35]

If $\Lambda \left(lm\right)=2\sqrt{A_{l}A_{m}}/A_{l}+A_{m}$, then

(10) $$\begin{array}{c} \underset{lm\in E\left(F\right)}{\sum }\Lambda \left(lm\right)=\underset{lm\in E\left(F\right)}{\sum }\frac{2\sqrt{A_{l}A_{m}}}{A_{l}+A_{m}}=GA_{5}\left(F\right)\text{.} \end{array}$$

Equation (1) is now changed to the following form, which is known as fifth geometric arithmetic entropy.

(11) $$\begin{array}{c} ENT_{GA_{5}}\left(F\right)=\left.\log\;\left(GA_{5}\left(F\right)\right)-\frac{1}{\left(GA_{5}\left(F\right)\right)}\overset{q}{\underset{i=1}{\sum }}\underset{lm\in E_{i}\left(F\right)}{\sum }\log{\left[\frac{2\sqrt{A_{l}A_{m}}}{A_{l}+A_{m}}\right]}^{\left[\frac{2\sqrt{A_{l}A_{m}}}{A_{l}+A_{m}}\right]}\right]\text{.} \end{array}$$

See [35, 44] for further information on these entropy measures.

## 2. Formation of Triangular Benzenoid *T*_*x*_∀ *x* ∈ *ℕ*

**Figure 1:**
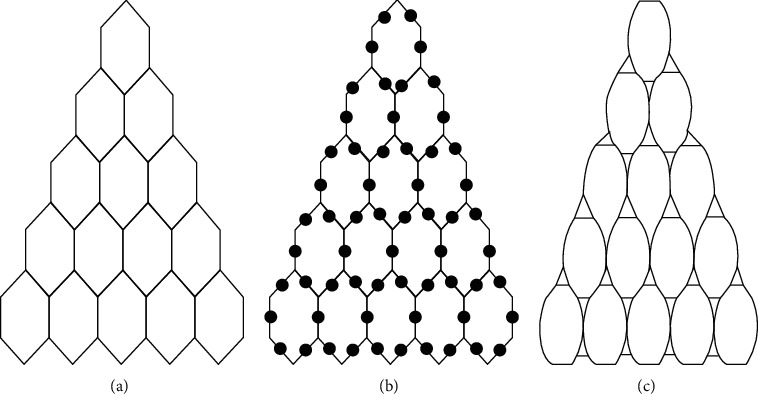
(a) Triangular benzenoid *T*_5_, (b) Subdivision of *T*_5_,  (c) The line graph of subdivision graph of *T*_5_.

Triangular benzenoids are a group of benzenoid molecular graphs and are denoted by *T*_*x*_, where *x* characterizes the number of hexagons at the bottom of the graph and 1/2*x*(*x*+1) represents the total number of hexagons in *T*_*x*_. Triangular benzenoids are a generalization of the benzene molecule *C*_6_*H*_6_, with benzene rings forming a triangular shape. In physics, chemistry, and nanosciences, the benzene molecule is a common molecule. Synthesizing aromatic chemicals is quite fruitful [46]. Raut [47] calculated some toplogical indices for the triangular benzenoid system. Hussain et al. [48] discussed the irregularity determinants of some benzenoid systems. Kwun [49] calculated degree-based indices by using *M* polynomials. For further details, see [50, 51]. The hexagons are placed in rows, with each row increasing by one hexagon. For *T*_1_, there are only one type of edges *e*_1_=(2,2) and |*e*_1_|=6. Therefore, *V*(*T*_1_)=6 and *E*(*T*_1_)=6 while three kinds of edges are there in *T*_2_ e.g. *e*_1_=(2,2), *e*_2_=(2,3), *e*_3_=(3,3) and |*e*_1_|=6, |*e*_2_|=6, |*e*_3_|=3. Therefore, *V*(*T* − 2)=13 and *E*(*T*_2_)=15. Continuing in this way, |*V*(*T*_*x*_)|=*x*^2^+4*x*+1 and |*E*(*T*_*x*_)|=3/2*x*(*x*+3). The subdivision graph of *T*_*x*_ and its line graph are demonstrated in Figure 1. It is to be noted that |*V*(*L*(*S*(*T*_*x*_)))|=3*x*(*x*+3) and |*E*(*L*(*S*(*T*_*x*_)))|=3/2(3*x*^2^+7*x* − 2).

Let *ℱ*=*L*(*S*(*T*_*x*_)). i-e. *ℱ* is the line graph of the subdivision graph of triangular benzenoid *T*_*x*_. We will use the edge partition and vertices counting technique to compute our abstracted indices and entropies. The degree of each edge's terminal vertices is used in the edge partitioning of *ℱ*. It is easy to see that there are only three types of edges shown in Table 1.

### 2.1. Entropy Measure for *L*(*S*(*T*_*x*_))

We'll calculate the entropies of *ℱ*=*L*(*S*(*T*_*x*_)) in this section.

#### 2.1.1. Randi$\overset{\prime }{c}$ Entropy of *L*(*S*(*T*_*x*_))

The Randi$\overset{\prime }{c}$ index and entropy for *α*=1, −1, 1/2, −1/2, with the help of Table 1, and equation (3) is:

(12) $$\begin{array}{c} ENT_{R_{\alpha }}\left(F\right)=\log\left(R_{\alpha }\right)-\frac{1}{\left(R_{\alpha }\right)}\overset{3}{\underset{i=1}{\sum }}\underset{lm\in E_{i}\left(F\right)}{\sum }\left[{\left(\overset{\wedge }{ℵ}\left(l\right)\times \overset{\wedge }{ℵ}\left(m\right)\right)}^{\alpha }\right]\log\left[{\left(\overset{\wedge }{ℵ}\left(l\right)\times \overset{\wedge }{ℵ}\left(m\right)\right)}^{\alpha }\right]\\ =\log\left(R_{\alpha }\right)-\frac{1}{\left(R_{\alpha }\right)}\left[\left[4^{\alpha }\left(3x+9\right)\times \log\left(4^{\alpha }\right)\right]+\left[6^{\alpha }\left(6x-6\right)\times \log\left(6^{\alpha }\right)\right]+\left[\frac{3^{\left(2\alpha +1\right)}}{2}\left(3x^{2}+x-4\right)\times \log\left(9^{\alpha }\right)\right]\right]\text{.} \end{array}$$

By putting *α*=1, −1, 1/2, −1/2, in (3), we get the Randi$\overset{\prime }{c}$ entropies as given below:

(13) $$\begin{array}{c} ENT_{R_{1}}\left(F\right)=\log\left(\frac{3}{2}\left(27x^{2}+41x-52\right)\right)-\frac{12\left(x+3\right)\times \log\left[4\right]}{\left(3/2\left(27x^{2}+41x-52\right)\right)}-\frac{36\left(x-1\right)\times \log\left[6\right]}{\left(3/2\left(27x^{2}+41x-52\right)\right)}-{\frac{27/2\left(3x^{2}+x-4\right)\times \log\left[9\right]}{\left(3/2\left(27x^{2}+41x-52\right)\right)}}^{\prime }\\ ENT_{R_{-1}}\left(F\right)=\log\;\left(\frac{1}{12}\left(6x^{2}+23x+7\right)\right)+\frac{3/4\left(x+3\right)\times \log\left[4\right]}{\left(1/12\left(6x^{2}+23x+7\right)\right)}+\frac{\left(x-1\right)\times \log\left[6\right]}{\left(1/12\left(6x^{2}+23x+7\right)\right)}+\frac{1/6\left(3x^{2}+x-4\right)\times \log\left[9\right]}{\left(1/12\left(6x^{2}+23x+7\right)\right)}\text{,}\\ ENT_{R_{1/2}}\left(F\right)=\log\;\left(\frac{3}{2}\left(9x^{2}+\left(7+4\sqrt{6}\right)x-4\sqrt{6}\right)\right)-\frac{6\left(x+3\right)\times \log\left[2\right]}{\left(3/2\left(9x^{2}+\left(7+4\sqrt{6}\right)x-4\sqrt{6}\right)\right)}-\frac{6\sqrt{6}\left(x-1\right)\times \log\left[\sqrt{6}\right]}{\left(3/2\left(9x^{2}+\left(7+4\sqrt{6}\right)x-4\sqrt{6}\right)\right)}-\frac{9/2\left(3x^{2}+x-4\right)\times \log\left[3\right]}{\left(3/2\left(9x^{2}+\left(7+4\sqrt{6}\right)x-4\sqrt{6}\right)\right)}\text{,}\\ ENT_{R_{-1/2}}\left(F\right)=\log\;\left(1/2\left(3x^{2}+2\left(2+\sqrt{6}\right)x+5\right)\right)+\frac{3/2\left(x+3\right)\times \log\left[2\right]}{\left(1/2\left(3x^{2}+2\left(2+\sqrt{6}\right)x+5\right)\right)}+\frac{\sqrt{6}\left(x-1\right)\times \log\left[\sqrt{6}\right]}{\left(1/2\left(3x^{2}+2\left(2+\sqrt{6}\right)x+5\right)\right)}+\frac{1/2\left(3x^{2}+x-4\right)\times \log\left[3\right]}{\left(1/2\left(3x^{2}+2\left(2+\sqrt{6}\right)x+5\right)\right)}\text{.} \end{array}$$

#### 2.1.2. The *ABC* Entropy of *L*(*S*(*T*_*x*_))

The *ABC* index and entropy measure with the help of Table 1 and equation (5) is:

(14) $$\begin{array}{c} ABC\left(F\right)=3x^{2}+\left(\frac{9}{\sqrt{2}}+1\right)x+\frac{3}{\sqrt{2}}-4\text{,}\\ ENT_{ABC}\left(F\right)=\log\;\left(ABC\right)-\frac{1}{\left(ABC\right)}\overset{3}{\underset{i=1}{\sum }}\underset{lm\in E_{i}\left(F\right)}{\sum }\left[\sqrt{\frac{\overset{\wedge }{ℵ}\left(l\right)+\overset{\wedge }{ℵ}\left(m\right)-2}{\overset{\wedge }{ℵ}\left(l\right)\times \overset{\wedge }{ℵ}\left(m\right)}}\right]\log\left[\sqrt{\frac{\overset{\wedge }{ℵ}\left(l\right)+\overset{\wedge }{ℵ}\left(m\right)-2}{\overset{\wedge }{ℵ}\left(l\right)\times \overset{\wedge }{ℵ}\left(m\right)}}\right]\\ =\log\;\left(3x^{2}+\left(\frac{9}{\sqrt{2}}+1\right)x+\frac{3}{\sqrt{2}}-4\right)+\frac{1/\sqrt{2}\left(9x+3\right)\times \log\left[\sqrt{2}\right]}{\left(3x^{2}+\left(9/\sqrt{2}+1\right)x+3/\sqrt{2}-4\right)}-\frac{\left(3x^{2}+x-4\right)\times \log\;\left[2/3\right]}{\left(3x^{2}+\left(9/\sqrt{2}+1\right)x+3/\sqrt{2}-4\right)}\text{.} \end{array}$$

#### 2.1.3. The Geometric Arithmetic Entropy of *L*(*S*(*T*_*x*_))

The *GA* index and entropy measure with the help of Table 1 and equation (7) is:

(15) $$\begin{array}{c} GA\left(F\right)=\frac{9}{2}x^{2}+\frac{3x}{10}\left(8\sqrt{6}+15\right)-\frac{3}{5}\left(4\sqrt{6}-5\right)\\ ENT_{GA}\left(F\right)=\log\;\left(GA\right)-\frac{1}{\left(GA\right)}\overset{3}{\underset{i=1}{\sum }}\underset{lm\in E_{i}\left(F\right)}{\sum }\left[\frac{2\sqrt{\overset{\wedge }{ℵ}\left(l\right)\times \overset{\wedge }{ℵ}\left(m\right)}}{\overset{\wedge }{ℵ}\left(l\right)+\overset{\wedge }{ℵ}\left(m\right)}\right]\log\left[\frac{2\sqrt{\overset{\wedge }{ℵ}\left(l\right)\times \overset{\wedge }{ℵ}\left(m\right)}}{\overset{\wedge }{ℵ}\left(l\right)+\overset{\wedge }{ℵ}\left(m\right)}\right]\\ =\log\;\left(\frac{9}{2}x^{2}+\left(\frac{24\sqrt{6}+45}{10}\right)x+\frac{15-12\sqrt{6}}{5}\right)-\frac{12\sqrt{6}/5\left(x-1\right)\times \log\;\left[2\sqrt{6}/5\right]}{\left(9/2x^{2}+\left(24\sqrt{6}+45/10\right)x+15-12\sqrt{6}/5\right)}. \end{array}$$

#### 2.1.4. The *ABC*_4_ Entropy of *L*(*S*(*T*_*x*_))

The edge partition of the graph *L*(*S*(*T*_*x*_)) is grounded on the degree addition of terminal vertices of every edge, as shown in Table 2.

After simple calculations, by using Table 2 subject to the condition that *x* ≠ 1, we get

(16) $$\begin{array}{c} ABC_{4}\left(F\right)=\frac{3\sqrt{6}}{2}+\frac{3\sqrt{7}}{\sqrt{5}}+\frac{6\sqrt{2}}{5}\left(x-2\right)+\left(\frac{3\sqrt{11}}{\sqrt{10}}+\frac{3\sqrt{14}}{8}+\frac{\sqrt{15}}{\sqrt{2}}\right)\left(x-1\right)+\frac{2}{3}\left(3x^{2}-5x+2\right)\text{.} \end{array}$$

By using (9), the *ABC*_4_ entropy as follows:

(17) $$\begin{array}{c} ENT_{ABC_{4}}\left(F\right)=\log\;\left(ABC_{4}\right)-\frac{1}{\left(ABC_{4}\right)}\overset{7}{\underset{i=1}{\sum }}\underset{lm\in E_{i}\left(F\right)}{\sum }\left[\sqrt{\frac{A_{l}+A_{m}-2}{A_{l}A_{m}}}\right]\log\left[\sqrt{\frac{A_{l}+A_{m}-2}{A_{l}A_{m}}}\right]\text{,}\\ ENT_{ABC_{4}}\left(F\right)=\log\;\left(ABC_{4}\right)-\frac{\left[3\sqrt{6}/2\right]\log\;\left[\sqrt{6}/4\right]}{\left(ABC_{4}\right)}-\frac{\left[3\sqrt{7}/\sqrt{5}\right]\log\;\left[\sqrt{7}/2\sqrt{5}\right]}{\left(ABC_{4}\right)}\\ -\frac{\left[6\sqrt{2}/5\right]\left(x-1\right)\log\;\left[2\sqrt{2}/5\right]}{\left(ABC_{4}\right)}-\frac{\left[3\sqrt{11}/\sqrt{10}\right]\left(x-1\right)\log\;\left[\sqrt{11}/2\sqrt{10}\right]}{\left(ABC_{4}\right)}\\ -\frac{\left[3\sqrt{14}/8\right]\left(x-1\right)\log\;\left[\sqrt{14}/8\right]}{\left(ABC_{4}\right)}-\frac{\left[\sqrt{15}/\sqrt{2}\right]\left(x-1\right)\log\;\left[\sqrt{15}/6\sqrt{2}\right]}{\left(ABC_{4}\right)}-\frac{2/3\left(3x^{2}-5x+2\right)\log\;\left[4/9\right]}{\left(ABC_{4}\right)}\text{.} \end{array}$$

If we consider *x*=1, Then $ABC_{4}\left(ℱ\right)=9\sqrt{6}/4$, and *ENT*_*ABC*_4__(*ℱ*)=2.1972.

#### 2.1.5. The *GA*_5_ Entropy of *L*(*S*(*T*_*x*_))

After some simple calculations, the *GA*_5_ index may be calculated using Table 2 under the constraint that *x* ≠ 1.

(18) $$\begin{array}{c} GA_{5}\left(F\right)=3+\frac{8\sqrt{5}}{3}+3x+\left(\frac{24\sqrt{10}}{13}+\frac{72\sqrt{2}}{17}+3\right)\left(x-1\right)+\frac{3}{2}\left(3x^{2}-5n+2\right). \end{array}$$

Therefore, (11), with Table 2 converted in the form:

(19) $$\begin{array}{c} ENT_{GA_{5}}\left(F\right)=\log\;\left(GA_{5}\right)-\frac{1}{\left(GA_{5}\right)}\overset{7}{\underset{i=1}{\sum }}\underset{lm\in E_{i}\left(F\right)}{\sum }\left[\sqrt{\frac{A_{l}+A_{m}-2}{A_{l}A_{m}}}\right]\log\left[\sqrt{\frac{A_{l}+A_{m}-2}{A_{l}A_{m}}}\right]\text{,}\\ ENT_{GA_{5}}\left(F\right)=\log\;\left(GA_{5}\right)-\frac{\left[8\sqrt{5}/3\right]\log\;\left[4\sqrt{5}/9\right]}{\left(GA_{5}\right)}-\frac{\left[24\sqrt{10}/13\right]\left(x-1\right)\log\;\left[4\sqrt{10}/13\right]}{\left(GA_{5}\right)}-\frac{\left[72\sqrt{2}/17\right]\left(x-1\right)\log\;\left[12\sqrt{2}/17\right]}{\left(GA_{5}\right)}\text{.} \end{array}$$

## 3. Formation of Hexagonal Parallelogram Nanotubes *H*(*x*, *y*), ∀*x*, *y* ∈ *ℕ*

**Figure 2:**
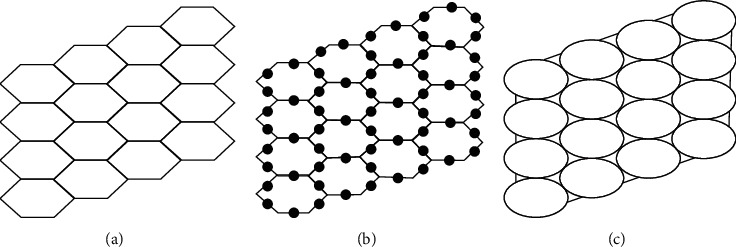
(a) Hexagonal parallelogram *H*(*x*, *y*), (b) Subdivision of *H*(*x*, *y*),  (c) The line graph of subdivision graph of *H*(*x*, *y*).

Hexagonal parallelogram nanotubes are formed by arranging hexagons in a parallelogram fashion. Baig et al. [52] computed counting polynomials of benzoid carbon nanotubes. Also, see [53]. We will denote this structure by *H*(*x*, *y*)∀ *x*, *y* ∈ *ℕ*, in which *x* and *y* represent the quantity of hexagons in any row and column respectively. Also, the order and size of *H*(*x*, *y*) is 2(*x*+*y*+*xy*) and 3*xy*+2*x*+2*y* − 1 respectively. The subdivision graph of *H*(*x*, *y*) and its line graph is shown in Figure 2, see [46]. Let *ℱ*=*L*(*S*(*H*(*x*, *y*))), then |*ℱ*_*V*_|=2(3*xy*+2*x*+2*y* − 1) and |*ℱ*_*E*_|=9*xy*+4*x*+4*y* − 5. To compute our results, we will use edge partition technique which is grounded on the degree of terminal vertices of every edge. It is to be noted that there are only three types of edges, see Figure 2. The edge partition of chemical graph *L*(*S*(*H*(*x*, *y*))) depending on the degree of terminal vertices is presented in Table 3.

### 3.1. Entropy Measure for *L*(*S*(*H*(*x*, *y*)))

We will enumerate the entropies of *ℱ*=*L*(*S*(*H*(*x*, *y*))) in this section.

#### 3.1.1. Randić Entropy of *ℱ*

The Randi$\overset{\prime }{c}$ index for *α*=1, −1, 1/2, −1/2, by using Table 3 is:

(20) $$\begin{array}{c} R_{\alpha }\left(F\right)=2\left(x+y+4\right)\times {\left(4\right)}^{\alpha }+4\left(x+y-2\right)\times {\left(6\right)}^{\alpha }+\left(9xy-2x-2y-5\right)\times {\left(9\right)}^{\alpha }\text{.} \end{array}$$

So the (3) with Table 3 gives the Randi$\overset{\prime }{c}$ entropy and is converted in the form:

(21) $$\begin{array}{c} ENT_{R_{\alpha }}\left(F\right)=\log\left(R_{\alpha }\right)-\frac{1}{\left(R_{\alpha }\right)}\overset{3}{\underset{i=1}{\sum }}\underset{lm\in E_{i}\left(F\right)}{\sum }\left[{\left(\overset{\wedge }{ℵ}\left(l\right)\times \overset{\wedge }{ℵ}\left(m\right)\right)}^{\alpha }\right]\log\left[{\left(\overset{\wedge }{ℵ}\left(l\right)\times \overset{\wedge }{ℵ}\left(m\right)\right)}^{\alpha }\right]\\ =\log\left(R_{\alpha }\right)-\frac{1}{\left(R_{\alpha }\right)}\left[\left[4^{\alpha }\left(2x+2y+8\right)\times \log\left(4^{\alpha }\right)\right]+\left[6^{\alpha }\left(4x+4y-8\right)\times \log\left(6^{\alpha }\right)\right]+\left[9^{\alpha }\left(9xy-2x-2y-5\right)\times \log\left(9^{\alpha }\right)\right]\right]\text{.} \end{array}$$

Now substitute *α*=1, −1, 1/2, −1/2, in (20), we get the Randi$\overset{\prime }{c}$ entropies as given below:

(22) $$\begin{array}{c} ENT_{R_{1}}\left(F\right)=\log\;\left(81xy+14\left(x+y\right)-61\right)-\frac{8\left(x+y+4\right)\times \left[4\right]}{\left(81xy+14\left(x+y\right)-61\right)}-\frac{24\left(x+y-2\right)\times \log\left[6\right]}{\left(81xy+14\left(x+y\right)-61\right)}-\frac{9\left(9xy-2x-2y-5\right)\times \log\left[9\right]}{\left(81xy+14\left(x+y\right)-61\right)}\\ ENT_{R_{-1}}\left(F\right)=\log\;\left(xy+\frac{17}{18}\left(x+y\right)+\frac{1}{9}\right)+\frac{1/2\left(x+y+4\right)\times \left[4\right]}{\left(xy+17/18\left(x+y\right)+1/9\right)}+\frac{2/3\left(x+y-2\right)\times \log\left[6\right]}{\left(xy+17/18\left(x+y\right)+1/9\right)}+\frac{1/9\left(9xy-2x-2y-5\right)\times \log\left[9\right]}{\left(xy+17/18\left(x+y\right)+1/9\right)}\text{.}\\ ENT_{R_{1/2}}\left(F\right)=\log\;\left(27xy+\left(4\sqrt{6}-2\right)\left(x+y\right)+1-8\sqrt{6}\right)-\frac{4\left(x+y+4\right)\times \left[2\right]}{\left(27xy+\left(4\sqrt{6}-2\right)\left(x+y\right)+1-8\sqrt{6}\right)}\\ -\frac{4\sqrt{6}\left(x+y-2\right)\times \log\left[\sqrt{6}\right]}{\left(27xy+\left(4\sqrt{6}-2\right)\left(x+y\right)+1-8\sqrt{6}\right)}-\frac{93\left(9xy-2x-2y-5\right)\times \log\left[3\right]}{\left(27xy+\left(4\sqrt{6}-2\right)\left(x+y\right)+1-8\sqrt{6}\right)}\text{,}\\ ENT_{R_{-1/2}}\left(F\right)=\log\;\left(3xy+\left(\frac{1}{3}+\frac{4}{\sqrt{6}}\right)\left(x+y\right)+\frac{7}{3}-\frac{8}{\sqrt{6}}\right)+\frac{\left(x+y+4\right)\times \log\left[2\right]}{\left(3xy+\left(1/3+4/\sqrt{6}\right)\left(x+y\right)+7/3-8/\sqrt{6}\right)}\\ +\frac{4/\sqrt{6}\left(x+y-2\right)\times \log\left[\sqrt{6}\right]}{\left(3xy+\left(1/3+4/\sqrt{6}\right)\left(x+y\right)+7/3-8/\sqrt{6}\right)}+\frac{1/3\left(9xy-2x-2y-5\right)\times \log\left[9\right]}{\left(3xy+\left(1/3+4/\sqrt{6}\right)\left(x+y\right)+7/3-8/\sqrt{6}\right)}\text{.} \end{array}$$

#### 3.1.2. The *ABC* Entropy of *ℱ*

With the use of Table 3 and equation (5), we can calculate the *ABC* index and entropy measure as follows:

(23) $$\begin{array}{c} ABC\left(F\right)=6xy+\left(\frac{9\sqrt{2}-4}{3}\right)\left(x+y\right)-\frac{10}{3}\text{.} \end{array}$$

Therefore, the equation (5), with Table 3 becomes as following and is called the atom bond connectivity entropy.

(24) $$\begin{array}{c} ENT_{ABC}\left(F\right)=\log\;\left(ABC\right)-\frac{1}{\left(ABC\right)}\overset{3}{\underset{i=1}{\sum }}\underset{lm\in E_{i}\left(F\right)}{\sum }\left[\sqrt{\frac{\overset{\wedge }{ℵ}\left(l\right)+\overset{\wedge }{ℵ}\left(m\right)-2}{\overset{\wedge }{ℵ}\left(l\right)\times \overset{\wedge }{ℵ}\left(m\right)}}\right]\log\left[\sqrt{\frac{\overset{\wedge }{ℵ}\left(l\right)+\overset{\wedge }{ℵ}\left(m\right)-2}{\overset{\wedge }{ℵ}\left(l\right)\times \overset{\wedge }{ℵ}\left(m\right)}}\right]\\ =\log\;\left(6xy+\left(\frac{9\sqrt{2}-4}{3}\right)\left(x+y\right)-\frac{10}{3}\right)+\frac{\sqrt{2}\left(x+y+4\right)\times \left[\sqrt{2}\right]}{\left(6xy+\left(9\sqrt{2}-4/3\right)\left(x+y\right)-10/3\right)}\\ +\frac{2\sqrt{2}\left(x+y-2\right)\times \log\left[\sqrt{6}\right]}{\left(6xy+\left(9\sqrt{2}-4/3\right)\left(x+y\right)-10/3\right)}-\frac{2/3\left(9xy-2x-2y-5\right)\times \log\;\left[2/3\right]}{\left(6xy+\left(9\sqrt{2}-4/3\right)\left(x+y\right)-10/3\right)}\text{.} \end{array}$$

#### 3.1.3. The Geometric Arithmetic Entropy of *ℱ*

We can calculate the *GA* index and entropy measure using Table 3 and equation (7) as follows:

(25) $$\begin{array}{c} GA\left(F\right)=\frac{1}{5}\left(45xy+8\sqrt{6}\left(x+y\right)+15-16\sqrt{6}\right)\text{,}\\ ENT_{GA}\left(F\right)=\log\;\left(\frac{1}{5}\left(45xy+8\sqrt{6}\left(x+y\right)+15-16\sqrt{6}\right)\right)-\frac{8\sqrt{6}/5\left(x+y-2\right)\times \log\;\left[2\sqrt{6}/5\right]}{\left(1/5\left(45xy+8\sqrt{6}\left(x+y\right)+15-16\sqrt{6}\right)\right)}\text{.} \end{array}$$

#### 3.1.4. The *ABC*_4_ Entropy of *ℱ*

**Case 1 .**

when *x* > 1, *y* ≠ 1

The edge partition of *L*(*S*(*H*(*x*, *y*))) is shown in Table 4.

Therefore, the *ABC*_4_ index and entropy measure with the help of Table 4 and equation (9) yield as:

(26) $$\begin{array}{c} ABC_{4}\left(F\right)=4xy+\left(\frac{4\sqrt{2}}{5}+\frac{2\sqrt{11}}{\sqrt{10}}+\frac{\sqrt{14}}{4}+\frac{\sqrt{30}}{3}-\frac{32}{9}\right)\left(x+y\right)+2\sqrt{6}+\frac{4\sqrt{7}}{\sqrt{5}}-\frac{16\sqrt{2}}{5}-\frac{4\sqrt{11}}{\sqrt{10}}-\frac{\sqrt{14}}{2}-\frac{2\sqrt{30}}{3}+\frac{28}{9}\text{.} \end{array}$$

Since *ℱ* has seven kinds of edges, So (9) by using Table 4 is converted in the form:

(27) $$\begin{array}{c} ENT_{ABC_{4}}\left(F\right)=\log\;\left(ABC_{4}\right)-\frac{1}{\left(ABC_{4}\right)}\overset{7}{\underset{i=1}{\sum }}\underset{lm\in E_{i}\left(F\right)}{\sum }\left[\sqrt{\frac{A_{l}+A_{m}-2}{A_{l}A_{m}}}\right]\log\left[\sqrt{\frac{A_{l}+A_{m}-2}{A_{l}A_{m}}}\right],\\ ENT_{ABC_{4}}\left(F\right)=\log\;\left(ABC_{4}\right)-\frac{2\sqrt{6}\log\;\left[\sqrt{6}/4\right]}{\left(ABC_{4}\right)}-\frac{4\sqrt{7}/\sqrt{5}\log\;\left[\sqrt{7}/2\sqrt{5}\right]}{\left(ABC_{4}\right)}\\ -\frac{4\sqrt{2}/5\left(x+y-4\right)\log\;\left[2\sqrt{2}/5\right]}{\left(ABC_{4}\right)}-\frac{2\sqrt{11}/\sqrt{10}\left(x+y-2\right)\log\;\left[\sqrt{11}/2\sqrt{10}\right]}{\left(ABC_{4}\right)}\\ -\frac{\sqrt{14}/4\left(x+y-2\right)\log\;\left[\sqrt{14}/8\right]}{\left(ABC_{4}\right)}-\frac{2\sqrt{15}/3\sqrt{2}\left(x+y-2\right)\log\;\left[\sqrt{15}/6\sqrt{2}\right]}{\left(ABC_{4}\right)}\\ -\frac{4/9\left(9xy-8x-8y+7\right)\log\;\left[4/9\right]}{\left(ABC_{4}\right)}\text{.} \end{array}$$

**Case 2 .**

when *x*=1, *y* ≠ 1

By using the same process, we get the closed expressions for the *ABC*_4_ index and *ABC*_4_ entropy as:

(28) $$\begin{array}{c} BC_{4}\left(F\right)=\left(\frac{4\sqrt{2}}{5}+\frac{2\sqrt{11}}{\sqrt{10}}+\frac{\sqrt{14}}{4}+\frac{\sqrt{30}}{3}+\frac{4}{9}\right)y+\frac{5\sqrt{6}}{2}+\frac{2\sqrt{7}}{\sqrt{5}}-\frac{8\sqrt{2}}{5}-\frac{2\sqrt{11}}{\sqrt{10}}-\frac{\sqrt{30}}{3}-\frac{\sqrt{14}}{4}-A\frac{9}{4}\text{,}\\ ENT_{ABC_{4}}\left(F\right)=\log\;\left(ABC_{4}\right)-\frac{5\sqrt{6}/2\log\;\left[\sqrt{6}/4\right]}{\left(ABC_{4}\right)}-\frac{2\sqrt{7}/\sqrt{5}\log\;\left[\sqrt{7}/2\sqrt{5}\right]}{\left(ABC_{4}\right)}-\frac{4\sqrt{2}/5\left(y-2\right)\log\;\left[2\sqrt{2}/5\right]}{\left(ABC_{4}\right)}\\ -\frac{2\sqrt{11}/\sqrt{10}\left(y-1\right)\log\;\left[\sqrt{11}/2\sqrt{10}\right]}{\left(ABC_{4}\right)}-\frac{\sqrt{14}/4\left(y-1\right)\log\;\left[\sqrt{14}/8\right]}{\left(ABC_{4}\right)}\\ -\frac{2\sqrt{15}/3\sqrt{2}\left(y-1\right)\log\;\left[\sqrt{15}/6\sqrt{2}\right]}{\left(ABC_{4}\right)}-\frac{4/9\left(y-1\right)\log\;\left[4/9\right]}{\left(ABC_{4}\right)}\text{.} \end{array}$$

#### 3.1.5. The Fifth Geometric Arithmetic Entropy of *ℱ*

**Case 3 .**

when *x* > 1, *y* ≠ 1The fifth geometric arithmetic entropy can be estimated by using (11), and Table 4 in the following manner:

(29) $$\begin{array}{c} GA_{5}\left(F\right)=9xy+\left(\frac{16\sqrt{10}}{13}+\frac{48\sqrt{2}}{17}-4\right)\left(x+y\right)+3+\frac{32\sqrt{5}}{9}-\frac{32\sqrt{10}}{13}-\frac{96\sqrt{2}}{17}\text{.} \end{array}$$

So the (11), with Table 4 can be written as:

(30) $$\begin{array}{c} ENT_{GA_{5}}\left(F\right)=\log\;\left(GA_{5}\right)-\frac{1}{\left(GA_{5}\right)}\overset{7}{\underset{i=1}{\sum }}\underset{lm\in E_{i}\left(F\right)}{\sum }\left[\sqrt{\frac{A_{l}+A_{m}-2}{A_{l}A_{m}}}\right]\log\left[\sqrt{\frac{A_{l}+A_{m}-2}{A_{l}A_{m}}}\right]\\ =\log\;\left(GA_{5}\right)-\frac{32\sqrt{5}/9\log\;\left[4\sqrt{5}/9\right]}{\left(GA_{5}\right)}-\frac{16\sqrt{10}/13\left(x+y-2\right)\log\;\left[4\sqrt{10}/13\right]}{\left(GA_{5}\right)}-\frac{48\sqrt{2}/17\left(x+y-2\right)\log\;\left[12\sqrt{2}/17\right]}{\left(GA_{5}\right)}\text{.} \end{array}$$

**Case 4 .**

when *x*=1, *y* ≠ 1By using Table 5 and using (11) we get the closed expressions for the *GA*_5_ index and *GA*_5_ entropy as:

(31) $$\begin{array}{c} GA_{5}\left(F\right)=\left(5+\frac{16\sqrt{10}}{13}+\frac{48\sqrt{2}}{17}\right)y+3+\frac{16\sqrt{5}}{9}-\frac{16\sqrt{10}}{13}-\frac{48\sqrt{2}}{17}-\frac{16\sqrt{10}}{13}\text{,}\\ ENT_{GA_{5}}\left(F\right)=\log\;\left(GA_{5}\right)-\frac{16\sqrt{5}/9\log\;\left[4\sqrt{5}/9\right]}{\left(GA_{5}\right)}-\frac{16\sqrt{10}/13\left(y-1\right)\log\;\left[4\sqrt{10}/13\right]}{\left(GA_{5}\right)}\\ -\frac{48\sqrt{2}/17\left(y-1\right)\log\;\left[12\sqrt{2}/17\right]}{\left(GA_{5}\right)}\text{.} \end{array}$$

## 4. Formation from Fusion of Zigzag-Edge Coronoid with Starphene *ZCS*(*x*, *y*, *z*) Nanotubes

**Figure 3:**
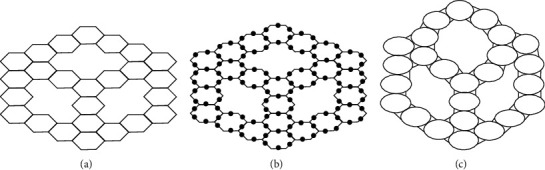
(a) *ZCS*(4,4,4), (b) subdivision of *ZCS*(4,4,4),  (c) *L*(*S*(*ZCS*(4,4,4))).

If a zigzag-edge coronoid *ZC*(*x*, *y*, *z*) is fused with a starphene *St*(*x*, *y*, *z*), then we will obtain a composite benzenoid. It is to *b* noted that |*V*(*ZCS*(*x*, *y*, *z*))|=36*x* − 54 and |*E*(*ZCS*(*x*, *y*, *z*))|=−63+15(*z*+*y*+*x*). The subdivision graph of *ZCS*(*x*, *y*, *z*) and its line graph are illustrated in Figure 3. We can see from figures that the order and the size in the line graph of the subdivision graph of *ZCS*(*x*, *y*, *z*) are −126+30(*z*+*y*+*x*) and −153+39(*z*+*y*+*x*) respectively [46]. Let *ℱ* represents the subdivision graph of *ZCS*(*x*, *y*, *z*)'s line graph. The edge division is determined by the degree of each edge's terminal vertices.  Table 6 illustrates this.

### 4.1. Entropy Measure for *L*(*S*(*ZCS*(*x*, *y*, *z*)))

We'll calculate the entropies of *ℱ*=*L*(*S*(*ZCS*(*x*, *y*, *z*))) in this section.

#### 4.1.1. Randi$\overset{\prime }{c}$ Entropy of *ℱ*

For *α*=1, −1, 1/2, −1/2, the Randi$\overset{\prime }{c}$ index with the help of Table 1 is

(32) $$\begin{array}{c} R_{\alpha }\left(F\right)=6\left(x+y+z-5\right)\times {\left(4\right)}^{\alpha }+12\left(x+y+z-7\right)\times {\left(6\right)}^{\alpha }+\left(21x+21y+21z-39\right)\times {\left(9\right)}^{\alpha }\text{.} \end{array}$$

Using (3) Randi$\overset{\prime }{c}$ entropy is:

(33) $$\begin{array}{c} ENT_{R_{\alpha }}\left(F\right)=\log\left(R_{\alpha }\right)-\frac{1}{\left(R_{\alpha }\right)}\overset{3}{\underset{i=1}{\sum }}\underset{lm\in E_{i}\left(F\right)}{\sum }\left[\left(\overset{\wedge }{ℵ}\left(l\right)\times \overset{\wedge }{ℵ}{\left(m\right)}^{\alpha }\right.\right]\log\;\left[\left(\overset{\wedge }{ℵ}\left(l\right)\times \overset{\wedge }{ℵ}{\left(m\right)}^{\alpha }\right.\right]\\ =\log\left(R_{\alpha }\right)-\frac{1}{\left(R_{\alpha }\right)}\left[\left[4^{\alpha }\left(6\left(x+y+z-5\right)\right)\times \log\left(4^{\alpha }\right)\right]+\left[6^{\alpha }\left(12\left(x+y+z-7\right)\right)\times \log\left(6^{\alpha }\right)\right]\right.\\ +\left.\left.\left[\left(21\left(x+y+z\right)-39\right)\times \log\left(9^{\alpha }\right)\right]\right]\right]\text{.} \end{array}$$

By putting *α*=1, −1, 1/2, −1/2, in (32), we get the Randi$\overset{\prime }{c}$ entropies as given below:

(34) $$\begin{array}{c} ENT_{R_{1}}\left(F\right)=\log\;\left(-975+285\left(z+y+x\right)\right)-\frac{24\left(-5+z+y+x\right)\times \log\left[4\right]}{\left(-975+285\left(z+y+x\right)\right)}-\frac{72\left(x+y+z-7\right)\times \log\left[6\right]}{\left(-975+285\left(z+y+x\right)\right)}\\ -\frac{189\left.\left(z+y+x\right)|-|351\right)\times \log\left[9\right]}{\left(-975+285\left(z+y+x\right)\right)}\text{,}\\ ENT_{R_{-1}}\left(F\right)=\log\;\left(-\frac{131}{6}+\frac{35}{6}\left(z+y+x\right)\right)+\frac{3/2\left(-5+z+y+x\right)\times \log\left[4\right]}{\left(-131/6+35/6\left(z+y+x\right)\right)}+\frac{2\left(-7+z+y+x\right)\times \log\left[6\right]}{\left(-131/6+35/6\left(z+y+x\right)\right)}\\ +\frac{1/3\left(-13+7\left(z+y+x\right)\right)\times \log\left[9\right]}{\left(-131/6+35/6\left(z+y+x\right)\right)}\text{,}\\ ENT_{R_{1/2}}\left(F\right)=\log\;\left(\left(3+12\sqrt{6}\right)\left(x+y+z\right)-177-84\sqrt{6}\right)-\frac{12\left(x+y+z-5\right)\times \log\left[2\right]}{\left(\left(3+12\sqrt{6}\right)\left(x+y+z\right)-177-84\sqrt{6}\right)}\\ -\frac{6\sqrt{6}\left(x+y+z-7\right)\times \log\left[\sqrt{6}\right]}{\left(\left(3+12\sqrt{6}\right)\left(x+y+z\right)-177-84\sqrt{6}\right)}-\frac{9\left(7\left(x+y+z\right)-13\right)\times \log\left[3\right]}{\left(\left(3+12\sqrt{6}\right)\left(x+y+z\right)-177-84\sqrt{6}\right)}\text{,}\\ ENT_{R_{-1/2}}\left(F\right)=\log\;\left(\left(10+2\sqrt{6}\right)\left(x+y+z\right)-28-14\sqrt{6}\right)+\frac{3\left(x+y+z-5\right)\times \log\left[2\right]}{\left(\left(10+2\sqrt{6}\right)\left(x+y+z\right)-28-14\sqrt{6}\right)}\\ +\frac{2\sqrt{6}\left(x+y+z-7\right)\times \log\left[\sqrt{6}\right]}{\left(\left(10+2\sqrt{6}\right)\left(x+y+z\right)-28-14\sqrt{6}\right)}+\frac{\left(7\left(x+y+z\right)-13\right)\times \log\left[3\right]}{\left(\left(10+2\sqrt{6}\right)\left(x+y+z\right)-28-14\sqrt{6}\right)}\text{.} \end{array}$$

#### 4.1.2. The *ABC* Entropy of *ℱ*

The *ABC* index and entropy measure with the help of Table 6 and equation (5) are:

(35) $$\begin{array}{c} ABC\left(F\right)=\left(14+9\sqrt{2}\right)\left(x+y+z\right)-26-57\sqrt{2}\text{,}\\ ENT_{ABC}\left(F\right)=\log\;\left(\left(14+9\sqrt{2}\right)\left(x+y+z\right)-26-57\sqrt{2}\right)+\frac{3\sqrt{2}\left(3\left(x+y+z\right)-19\right)\times \log\left[\sqrt{2}\right]}{\left(\left(14+9\sqrt{2}\right)\left(x+y+z\right)-26-57\sqrt{2}\right)}\\ -\frac{\left(14\left(x+y+z\right)-26\right)\log\;\left[2/3\right]}{\left(\left(14+9\sqrt{2}\right)\left(x+y+z\right)-26-57\sqrt{2}\right)}\text{.} \end{array}$$

#### 4.1.3. The Geometric Arithmetic Entropy of *ℱ*

The *GA* index and corresponding entropy with the help of Table 6 and equation (7) are:

(36) $$\begin{array}{c} GA\left(F\right)=\left(x+y+z\right)\left(27+24\sqrt{6}/5\right)-69-168\sqrt{6}/5\text{,}\\ ENT_{GA}\left(F\right)=\log\left(\left(x+y+z\right)\left(27+24\sqrt{6}/5\right)-69-168\sqrt{6}/5\right)-\frac{24\sqrt{6}/5\left(x+y+z-7\right)\times \log\;\left[2\sqrt{6}/5\right]}{\left(x+y+z\right)\left(27+24\sqrt{6}/5\right)-69-168\sqrt{6}/5}\text{.} \end{array}$$

#### 4.1.4. The *ABC*_4_ entropy of *ℱ*

Table 7 shows the graph *L*(*S*(*ZCS*(*x*, *y*, *z*)))'s edge partition, which is based on the degree addition of each edge's terminal vertices.

After simple calculations, the *ABC*_4_ index and entropy measure with the help of Table 7 and equation (9) subject to the condition that *x*=*y*=*z* ≥ 41

(37) $$\begin{array}{c} ABC_{4}\left(F\right)=\left(x+y+z\right)\left(\sqrt{30}+\frac{4}{3}+\frac{12\sqrt{2}}{5}+\frac{3\sqrt{14}}{4}+\frac{6\sqrt{11}}{\sqrt{10}}\right)-5\sqrt{30}+\frac{100}{3}-\frac{96\sqrt{2}}{5}+\frac{27\sqrt{14}}{4}-\frac{42\sqrt{11}}{\sqrt{10}}+\frac{3\sqrt{6}}{2}+\frac{6\sqrt{7}}{\sqrt{5}}\text{,}\\ ENT_{ABC_{4}}\left(F\right)=\log\;\left(ABC_{4}\right)-\frac{1}{\left(ABC_{4}\right)}\overset{7}{\underset{i=1}{\sum }}\underset{lm\in E_{i}\left(F\right)}{\sum }\left[\sqrt{\frac{A_{l}+A_{m}-2}{A_{l}A_{m}}}\right]\log\left[\sqrt{\frac{A_{l}+A_{m}-2}{A_{l}A_{m}}}\right],\\ ENT_{ABC_{4}}\left(F\right)=\log\;\left(ABC_{4}\right)-\frac{\left[3\sqrt{6}/2\right]\log\;\left[\sqrt{6}/4\right]}{\left(ABC_{4}\right)}-\frac{\left[12\sqrt{2}/5\right]\left(x+y+z-8\right)\log\;\left[2\sqrt{2}/5\right]}{\left(ABC_{4}\right)}\\ -\frac{\left[6\sqrt{11}/\sqrt{10}\right]\left(x+y+z-7\right)\log\;\left[\sqrt{11}/2\sqrt{10}\right]}{\left(ABC_{4}\right)}-\frac{3\sqrt{14}/4\left(x+y+x-9\right)\log\;\left[\sqrt{14}/8\right]}{\left(ABC_{4}\right)}\;\\ -\frac{\sqrt{30}\left(x+y+z-5\right)\log\;\left[\sqrt{15}/6\sqrt{2}\right]}{\left(ABC_{4}\right)}-\frac{4/3\left(x+y+z+25\right)\log\;\left[4/9\right]}{\left(ABC_{4}\right)}-\frac{\left[6\sqrt{7}/\sqrt{5}\right]\log\;\left[\sqrt{7}/2\sqrt{5}\right]}{\left(ABC_{4}\right)}\text{.} \end{array}$$

#### 4.1.5. The *GA*_5_ Entropy of *ℱ*

After some simple calculations, the *GA*_5_ index and corresponding entropy measure with the help of Table 7 and equation (11) subject to the condition that *x*=*y*=*z* ≥ 4.

(38) $$\begin{array}{c} GA_{5}\left(F\right)=\left(x+y+z\right)\left(15+\frac{48\sqrt{10}}{13}+\frac{144\sqrt{2}}{17}\right)+3+\frac{16\sqrt{5}}{3}-\frac{336\sqrt{10}}{13}-\frac{720\sqrt{2}}{17}\\ ENT_{GA_{5}}\left(F\right)=\log\;\left(GA_{5}\right)-\frac{1}{\left(GA_{5}\right)}\overset{7}{\underset{i=1}{\sum }}\underset{lm\in E_{i}\left(F\right)}{\sum }\left[\sqrt{\frac{A_{l}+A_{m}-2}{A_{l}A_{m}}}\right]\log\left[\sqrt{\frac{A_{l}+A_{m}-2}{A_{l}A_{m}}}\right],\\ ENT_{GA_{5}}\left(F\right)=\log\;\left(GA_{5}\right)-\frac{\left[16\sqrt{5}/3\right]\log\;\left[4\sqrt{5}/9\right]}{\left(GA_{5}\right)}-\frac{\left[48\sqrt{10}/13\right]\left(x+y+z-7\right)\log\;\left[4\sqrt{10}/13\right]}{\left(GA_{5}\right)}\\ -\frac{\left[144\sqrt{2}/17\right]\left(x+y+z-5\right)\log\;\left[12\sqrt{2}/17\right]}{\left(GA_{5}\right)}\text{.} \end{array}$$

## 5. Concluding remarks for Computed Results

The applications of information-theoretic framework in many disciplines of study, such as biology, physics, engineering, and social sciences, have grown exponentially in the recent two decades. This phenomenal increase has been particularly impressive in the fields of soft computing, molecular biology, and information technology. As a result, the scientists may find our numerical and graphical results useful [54, 55]. The entropy function is monotonic, which means that as the size of a chemical structure increases, so does the entropy measure, and as the entropy of a system increases, so does the uncertainty regarding its reaction.

**Figure 4:**
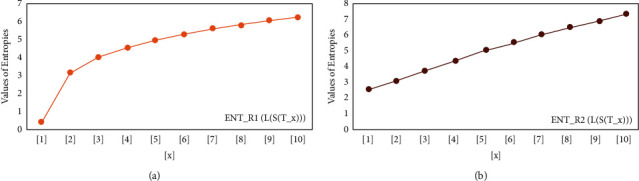
(a) *R*_1_ entropy, (b) *R*_−1_ entropy.

**Figure 5:**
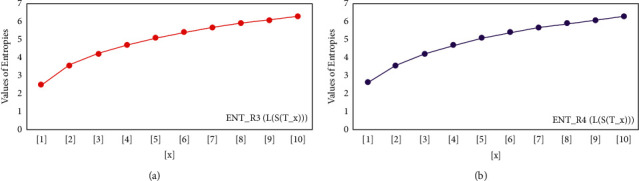
(a) *R*_1/2_ entropy, (b) *R*_−1/2_ entropy.

**Figure 6:**
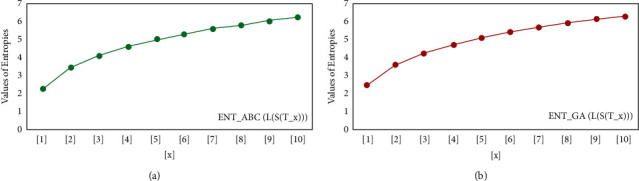
(a) The *ABC* entropy, (b) The *GA* entropy.

**Figure 7:**
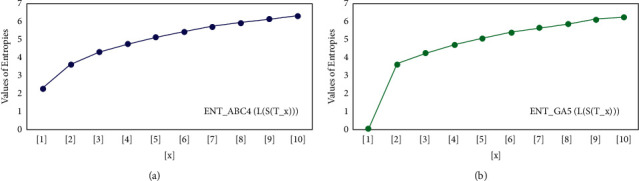
(a) The *ABC*_4_ entropy, (b) The *GA*_5_, entropy.

**Figure 8:**
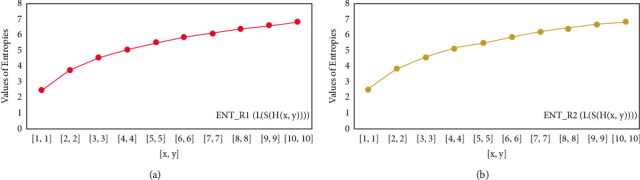
(a)*R*_1_ entropy, (b)*R*_−1_ entropy.

**Figure 9:**
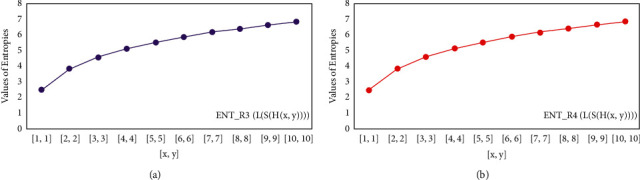
(a)*R*_1/2_ entropy, (b)*R*_−1/2_ entropy.

**Figure 10:**
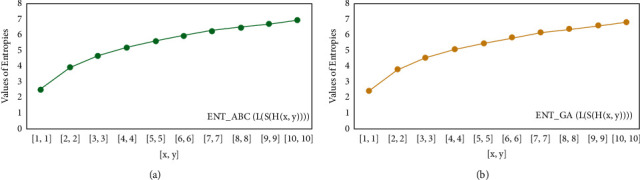
(a) The *ABC* entropy, (b) The *GA*, entropy.

**Figure 11:**
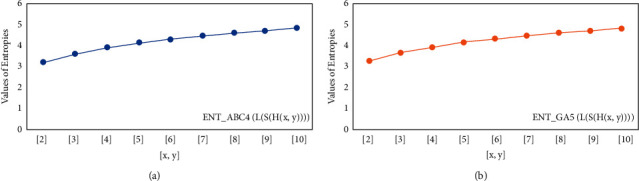
(a) The *ABC*_4_ entropy, (b) The *GA*_5_ entropy, *x* ≥ 1, *y* ≠ 1.

**Figure 12:**
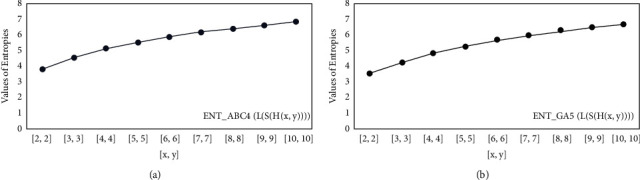
(a) The *ABC*_4_ entropy, (b) The *GA*_5_ entropy *x*=1, *y* ≠ 1.

**Figure 13:**
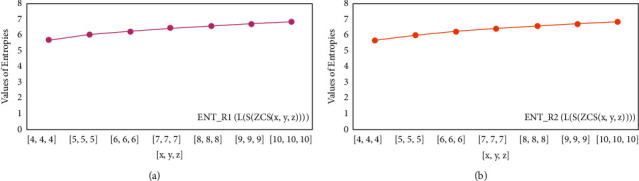
(a)*R*_1_ entropy, (b)*R*_−1_ entropy.

**Figure 14:**
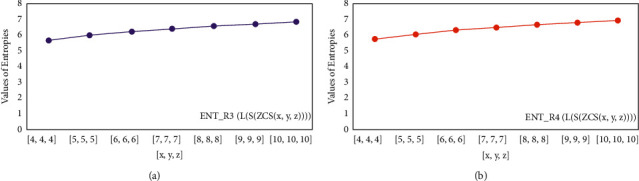
(a)*R*_1/2_ entropy, (b)*R*_1/2_ entropy.

**Figure 15:**
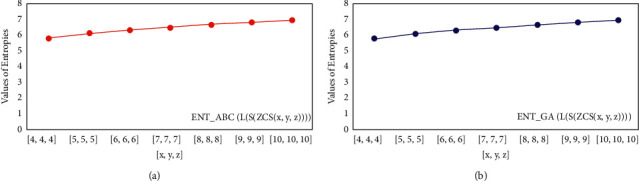
(a)*ABC* entropy, (b)The *GA*, entropy.

**Figure 16:**
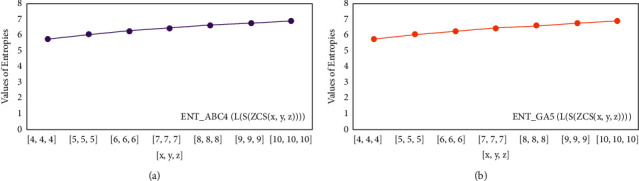
(a)*ABC*_4_ entropy, (b)*GA*_5_ entropy.

For *L*(*S*(*T*_*x*_)), the numerical and graphical results are shown in Tables 8 and 9 and Figures 4–7. In Table 9, the fifth arithmetic geometric entropy is zero which shows that the process is deterministic for *x*=1. When the chemical structure *L*(*S*(*T*_*x*_)) expands, the Randi$\overset{\prime }{c}$ entropy for *α*=1/2 develops more quickly than other entropy measurements of *L*(*S*(*T*_*x*_)), whereas the Randi$\overset{\prime }{c}$ entropy for *α*=−1/2 develops more slowly. This demonstrates that different topologies have varied entropy characteristics. For *L*(*S*(*H*(*x*, *y*))), the numerical and graphical results are shown in Tables 10–13 and Figures 8–12. When the chemical structure *L*(*S*(*H*(*x*, *y*))) expands, the geometric arithmetic entropy develops more quickly than other entropy measurements of *L*(*S*(*H*(*x*, *y*))), whereas the *ABC*_4_ entropy develops more slowly. Finally, for *L*(*S*(*ZCS*(*x*, *y*, *z*))), the numerical and graphical results are shown in Table 14 and Figures 13–16. When the chemical structure *L*(*S*(*ZCS*(*x*, *y*, *z*))) expands, the geometric arithmetic entropy develops more quickly than other entropy measurements of *L*(*S*(*ZCS*(*x*, *y*, *z*))), whereas the Randi$\overset{\prime }{c}$ entropy for *α*=−1 develops more slowly.

The novelty of this article is that entropies are computed for three types of benzenoid systems. These entropy measures are useful in estimating the heat of formation and many Physico-chemical properties. In statistical analysis of benzene structures, entropy measures showed more significant results as compared to topological indices. Therefore, we can say that the entropy measure is a newly introduced topological descriptor.

## 6. Conclusion

Using Shanon's entropy and Chen et al. [31] entropy definitions, we generated graph entropies associated to a new information function in this research. Between indices and information entropies, a relationship is created. Using the line graph of the subdivision of these graphs, we estimated the entropies for triangular benzenoids *T*_*x*_, hexagonal parallelogram *H*(*x*, *y*) nanotubes, and *ZCS*(*x*, *y*, *z*). Thermodynamic entropy of enzyme-substrate complexions [57, 58] and configuration entropy of glass-forming liquids [56] are two examples of thermodynamic entropy employed in molecular dynamics studies of complex chemical systems. Similarly, using information entropy as a crucial structural criterion could be a new step in this direction.

## Figures and Tables

**Table 1:** Edge partition of *L*(*S*(*T*_*x*_)).

$\left(\overset{\wedge }{ℵ}\left(l\right),\overset{\wedge }{ℵ}\left(m\right)\right)$	*N*_*i*_	Set of Edges
(2,2)	2(*x*+3)	*E*_1_
(2,3)	6(*x* − 1)	*E*_2_
(3,3)	3/2(3*x*^2^+*x* − 4)	*E*_3_

**Table 2:** Edge partition of *L*(*S*(*T*_*x*_)).

(*A*_*l*_, *A*_*m*_)	*N*_*i*_	Set of Edges
(4, 4)	9	*ℱ*_*E*_1__
(4, 5)	6	*ℱ*_*E*_2__
(5, 5)	3(*x* − 2)	*ℱ*_*E*_3__
(5, 8)	6(*y* − 1)	*ℱ*_*E*_4__
(8, 8)	3(*x* − 1)	*ℱ*_*E*_5__
(8, 9)	6(*x* − 1)	*ℱ*_*E*_6__
(9, 9)	3/2(3*x*^2^+2 − 5*x*)	*ℱ*_*E*_7__

**Table 3:** Edge partition of *L*(*S*(*H*(*x*, *y*))).

$\left(\overset{\wedge }{ℵ}\left(l\right),\overset{\wedge }{ℵ}\left(m\right)\right)$	*N*_*i*_	Kinds of Edges
(2, 2)	2(4+*y*+*x*)	*ℱ*_*E*_1__
(2, 3)	4(−2+*y*+*x*)	*ℱ*_*E*_2__
(3, 3)	9*xy* − 2*m* − 2*n* − 5	*ℱ*_*E*_3__

**Table 4:** Edge partition of *L*(*S*(*H*(*x*, *y*))).

(*A*_*l*_, *A*_*m*_)	*N*_*i*_	Kinds of edges
(4, 4)	8	*ℱ*_*E*_1__
(4, 5)	8	*ℱ*_*E*_2__
(5, 5)	2(−4+*y*+*x*)	*ℱ*_*E*_3__
(5, 8)	4(−2+*y*+*x*)	*ℱ*_*E*_4__
(8, 8)	2(−2+*x*+*y*)	*ℱ*_*E*_5__
(8, 9)	2(−2+*x*+*y*)	*ℱ*_*E*_6__
(9, 9)	9*xy* − 8*x* − 8*y*+7	*ℱ*_*E*_7__

**Table 5:** Edge partition of *L*(*S*(*H*(*x*, *y*))), for *x*=1.

(*A*_*l*_, *A*_*m*_)	*N*_*i*_	Kinds of edges
(4, 4)	10	*ℱ*_*E*_1__
(4, 5)	4	*ℱ*_*E*_2__
(5, 5)	2(*y* − 2)	*ℱ*_*E*_3__
(5, 8)	4(*y* − 1)	*ℱ*_*E*_4__
(8, 8)	2(*y* − 1)	*ℱ*_*E*_5__
(8, 9)	2(*y* − 1)	*ℱ*_*E*_6__
(9, 9)	*y* − 1	*ℱ*_*E*_7__

**Table 6:** Edge partition of *L*(*S*(*ZCS*)).

$\left(\overset{\wedge }{ℵ}\left(l\right),\overset{\wedge }{ℵ}\left(m\right)\right)$	*N*_*i*_	Kinds of Edges
(2,2)	6(−5+*z*+*y*+*x*)	*ℱ*_*E*_1__
(2,3)	12(−7+*z*+*y*+*x*)	*ℱ*_*E*_2__
(3,3)	−39+21(*z*+*y*+*x*)	*ℱ*_*E*_3__

**Table 7:** Edge partition of *L*(*S*(*ZCS*(*x*, *y*, *z*))) established on degree sum of terminal vertices, for every *x*=*y*=*z* ≥ 4

(*A*_*l*_, *A*_*m*_)	*N*_*i*_	Kinds of Edges
(4, 4)	6	*ℱ*_*E*_1__
(4, 5)	12	*ℱ*_*E*_2__
(5, 5)	6(*x*+*y*+*z* − 8)	*ℱ*_*E*_3__
(5, 8)	12(*x*+*y*+*z* − 7)	*ℱ*_*E*_4__
(8, 8)	6(*x*+*y*+*z* − 9)	*ℱ*_*E*_5__
(8, 9)	12(*x*+*y*+*z* − 5)	*ℱ*_*E*_6__
(9, 9)	3(*x*+*y*+*z*+25)	*ℱ*_*E*_7__

**Table 8:** Comparison of randic entropies for *L*(*S*(*T*_*x*_)).

[*x*]	*ENT*_*R*_1__	*ENT*_*R*_−1__	*ENT*_*R*_1/2__	*ENT*_*R*_−1/2__
[46]	0.4055	2.5590	2.4849	2.6263
[52]	3.1863	3.0463	3.5667	3.5970
[25]	4.0316	3.6767	4.2203	4.2280
[26]	4.5797	4.2928	4.6981	4.6991
[24]	4.9945	4.8714	5.0779	5.0764
[23]	5.3312	5.4107	5.3942	5.3918
[27]	5.6159	5.9131	5.6658	5.6631
[2]	5.8632	6.3820	5.9041	5.9013
[56]	6.0820	6.8208	6.1164	6.1136
[31]	6.2785	7.2325	6.3080	6.3053

**Table 9:** Comparison of *ENT*_*ABC*_, *ENT*_*GA*_, *ENT*_*ABC*_4__, and *ENT*_*GA*_5__ for *L*(*S*(*T*_*x*_)).

[*x*]	*ENT*_*ABC*_	*ENT*_*GA*_	*ENT*_*ABC*_4__	*ENT*_*GA*_5__
[46]	2.3116	2.4849	2.1972	0
[52]	3.5239	3.5835	3.5749	3.5835
[25]	4.2025	4.2341	4.2263	4.2341
[26]	4.6897	4.7095	4.7028	4.7095
[24]	5.0739	5.0876	5.0817	5.0876
[23]	5.3926	5.4027	5.3975	5.4026
[27]	5.6655	5.6733	5.6687	5.6733
[2]	5.9046	5.91087	5.9066	5.9108
[56]	6.1174	6.1225	6.1187	6.1225
[31]	6.3093	6.3135	6.3100	6.3135

**Table 10:** Comparison of randic entropies for *L*(*S*(*H*(*x*, *y*))).

[*x*, *y*]	*ENT*_*R*_1__	*ENT*_*R*_−1__	*ENT*_*R*_1/2__	*ENT*_*R*_−1/2__
[1,1]	2.4849	2.4849	2.4849	2.4849
[2,2]	3.7917	3.7830	3.8344	3.8332
[3,3]	4.5635	4.5428	4.5933	4.5906
[4,4]	5.1096	5.0872	5.1323	5.1294
[5,5]	5.5345	5.5129	5.5530	5.5502
[6,6]	5.8833	5.8630	5.8988	5.8962
[7,7]	6.1794	6.1615	6.1928	6.1904
[8,8]	6.4368	6.4194	6.4486	6.4464
[9,9]	6.6646	6.6483	6.6751	6.6731
[10,10]	6.8688	6.5370	6.8783	6.8822

**Table 11:** Comparison of *ENT*_*ABC*_ and *ENT*_*GA*_ entropies for *L*(*S*(*H*(*x*, *y*))).

[*x*, *y*]	*ENT*_*ABC*_	*ENT*_*GA*_
[1,1]	2.4849	2.4849
[2,2]	3.8497	3.8501
[3,3]	4.6048	4.6051
[4,4]	5.1413	5.1416
[5,5]	5.5604	5.5607
[6,6]	5.9051	5.9053
[7,7]	6.1982	6.1985
[8,8]	6.4534	6.4536
[9,9]	6.6794	6.6796
[10,10]	6.8822	6.8824

**Table 12:** Comparison of *ENT*_*ABC*_4__ and *ENT*_*GA*_5__ Entropies for *L*(*S*(*H*(*x*, *y*))), *x* > 1 and *y* ≠ 1.

[*x*, *y*]	*ENT*_*ABC*_4__	*ENT*_*GA*_5__
[2,2]	3.7879	3.4822
[3,3]	4.5387	2.2596
[4,4]	5.0783	4.8387
[5,5]	5.5018	5.2952
[6,6]	5.8509	5.6704
[7,7]	6.1481	5.9882
[8,8]	6.4068	6.2636
[9,9]	6.6360	6.5064
[10,10]	6.8417	6.7234

**Table 13:** Comparison of *ENT*_*ABC*_4__ and *ENT*_*GA*_5__ entropies for *L*(*S*(*H*(*x*, *y*))), *x*=1 and *y* ≠ 1.

[*y*]	*ENT*_*ABC*_4__	*ENT*_*GA*_5__
[52]	3.1846	3.2958
[25]	3.5933	3.6888
[26]	3.8884	3.9702
[24]	4.1184	4.1896
[23]	4.3064	4.3694
[27]	4.4653	4.5217
[2]	4.6027	4.6539
[56]	4.7238	4.7706
[31]	4.8319	4.8751

**Table 14:** Comparison of randic entropies for *L*(*S*(*ZCS*(*x*, *y*, *z*))).

[*x*, *y*, *z*]	*ENT*_*R*_1__	*ENT*_*R*_−1__	*ENT*_*R*_1/2__	*ENT*_*R*_−1/2__
[4,4,4]	5.7200	5.70060	5.7432	5.7407
[5,5,5]	6.0342	6.0165	6.0587	6.0565
[6,6,6]	6.2730	6.2564	6.2982	6.2961
[7,7,7]	6.4657	6.4497	6.4913	6.4893
[8,8,8]	6.6272	6.6117	6.6531	6.6511
[9,9,9]	6.7662	6.7511	6.7923	6.7904
[10,10,10]	6.8883	6.8734	6.9145	6.9126

## Data Availability

The data used to support the findings of this study are cited at relevant places within the text as references.
